# A Novel Monopulse Technique for Adaptive Phased Array Radar

**DOI:** 10.3390/s17010116

**Published:** 2017-01-08

**Authors:** Xinyu Zhang, Yang Li, Xiaopeng Yang, Le Zheng, Teng Long, Christopher J. Baker

**Affiliations:** 1 Department of Information and Electronics Engineering, Beijing Institute of Technology, Beijing 100081, China; zhangxinyu90111@gmail.com (X.Z.); xiaopengyang@bit.edu.cn (X.Y.); longteng@bit.edu.cn (T.L.); 2 School of Electrical Engineering, Columbia University, New York, NY 10027, USA; lezheng8451@163.com; 3 ElectroScience Laboratory, Ohio State University, Columbus, OH 43212, USA; baker.1891@osu.edu; 4 Beijing Key Laboratory of Embedded Real-time Information Processing Technology, Beijing Institute of Technology, Beijing 100081, China

**Keywords:** monopulse technique, adaptive phased array, interferences

## Abstract

The monopulse angle measuring technique is widely adopted in radar systems due to its simplicity and speed in accurately acquiring a target’s angle. However, in a spatial adaptive array, beam distortion, due to adaptive beamforming, can result in serious deterioration of monopulse performance. In this paper, a novel constrained monopulse angle measuring algorithm is proposed for spatial adaptive arrays. This algorithm maintains the ability to suppress the unwanted signals without suffering from beam distortion. Compared with conventional adaptive monopulse methods, the proposed algorithm adopts a new form of constraint in forming the difference beam with the merit that it is more robust in most practical situations. At the same time, it also exhibits the simplicity of one-dimension monopulse, helping to make this algorithm even more appealing to use in adaptive planar arrays. The theoretical mean and variance of the proposed monopulse estimator is derived for theoretical analysis. Mathematical simulations are formulated to demonstrate the effectiveness and advantages of the proposed algorithm. Both theoretical analysis and simulation results show that the proposed algorithm can outperform the conventional adaptive monopulse methods in the presence of severe interference near the mainlobe.

## 1. Introduction

Monopulse is an established technique in radars for fast and precise estimation of direction of target [1,2]. Compared with other precise angle estimation methods, such as MUSIC, maximum likelihood (ML) estimation etc., it consumes much less time and demands much lower computation cost. It can take only a single snapshot to make a precise estimation while its competitors usually have to solve a non-linear optimization problem [3,4]. In the case of a single target, it is an approximate ML approach, as shown in [5]. However, monopulse technique is not applicable in the case of multiple targets. Large aperture radar nowadays can easily avoid the case of multiple targets with narrower beam width. Modern wide band radars can also employ this technique [6]. Thus, the monopulse technique has still been a promising candidate for angle estimation in modern radar systems. Historically, the monopulse technique is implemented by either constructing error voltages or phase angles from a two-antenna system exploiting a fixed functional relation with the angle of target [7]. In a typical monopulse system, two sets of identical antennas are either separated by some distance (phase monopulse) or located at the the same phase center but with a squint angle difference (amplitude monopulse). Their outputs are summed up to produce a sum beam and are subtracted yielding the difference beam. When it comes to two dimensions, the system needs four receiving channels placed in four quadrants symmetrically to measure azimuth and elevation angles separately. With the advent of phased array radar, operation in the element level is impractical due to the large number of elements. Thus, the monopulse technique is usually implemented with digitalized sub-array outputs. When interference is present, adaptive beamforming [8] is required to suppress the unwanted signals when forming sum and difference beams. More recently, the method of adaptive beamforming based on artificial neural network has been presented in [9].

However, adaptive beamforming changes the beam pattern dependent on the received signals and the desired direction for nulling out the unwanted signals while maximizing the gain in the desired direction. This change results in a deviation in the pointing direction [10] and deformation of the main beam which in turn deteriorates angle measurement accuracy. Additionally, the distortion of the beam pattern is a function of the external environment, such as the location of the interference, target signal energy, clutter energy, etc. [7].

To alleviate distortion in the sum beam, a technique called “diagonal loading” has been introduced [11,12,13]. This technique, in general, makes a compromise between beam distortion and nulling depth by adding a diagonal matrix to the data covariance matrix. It has been proved that diagonal loading is mathematically equivalent to adding constraints about the pointing direction when forming sum beam weights [12]. By choosing a proper loading coefficient, the shape of the main beam can be maintained to be approximately the same while imposing sufficient suppression of the interference.

However, beam distortion may be more severe in forming the difference beam where the “diagonal loading” technique is no longer applicable. The solution to this problem requires keeping sufficient null depth in the direction of interference and maintaining a constant monopulse ratio. Methods based on the ML estimator have been developed in [14,15,16,17] and generalized in [7]. These methods are also referred to as adaptive corrected monopulse as they correct the distortion and bias in the monopulse ratio formula. A different minimum variance adaptive monopulse (MVAM) solution was proposed in [18] and it was found that this solution is equivalent to the ML estimator when the sum beam weight is derived for optimal signal-to-noise ratio (SNR). All these methods have been extended to the space-time case. This has been presented in [19,20,21]. However, all these methods require calculation of first or second derivatives of array manifold with respect to angles of arrival, which makes these methods more sensitive to the error of array elements distribution. Furthermore, these derivatives are calculated at the radar look direction to approximate its true value at the unknown target direction, which would result in performance deterioration. Other ML-based estimation schemes for multi-dimensional parameter estimation were introduced in [22,23], but the solutions are non-linear and ignore the correlation between azimuth and elevation estimates [7]. Another approach to the problem is by adding additional constraints to clamp the values of monopulse ratio at several selected direction [24,25,26]. These constraints work by trading degrees of freedom (DOF) for the desired difference beam pattern around the look direction. The loss of DOF reduces the output signal to noise and interference ratio (SNIR). However, how to choose these constraints to optimize the trade-off remains an open question. Furthermore, the constraints used in [24] ignore the fact that output of the beamforming process in most of the radar systems is actually complex for the sake of measuring the Doppler effect. As a result, the constraints imposed may consume more DOF and lead to further losses in SNIR.

In this paper, a new constrained monopulse algorithm is proposed to calculate adaptive difference beam weights. By using the proposed algorithm, unnecessary loss of DOF is saved, thus improving the performance of monopulse angle measurement. The proposed algorithm is then extended to planar array application, in which the estimation of azimuth and elevation is correlated with each other. Therefore, decoupling constraint is added to the constrained problem, making azimuth and elevation estimation independent from each other and the two-dimensional estimation can be solved by two separate one-dimensional monopulse measurements. The theoretical mean and variance of the proposed monopulse estimator is then derived for analysis using the distribution of the monopulse ratio and the optimized parameters in the constraints are also derived. Theoretical analysis shows that the estimates using the proposed algorithm are unbiased and comparison with the MVAM solution shows that the proposed algorithm can achieve better performance with optimized parameters. In addition, for the first time, we demonstrated that the MVAM could be considered as a special case of the linear constraint monopulse technique. Numerical simulation is employed to demonstrate the effectiveness and advantage of the proposed algorithm and the theoretical variance derived fits the simulation result perfectly well.

The remainder of this paper is organized as follows: in Section 2, a data model for the adaptive monopulse technique is outlined. The proposed algorithm is introduced and a closed form solution generating the adaptive difference beam weights is derived in Section 3.1. The algorithm is then extended to the case of planar arrays in Section 3.2. In Section 4, the theoretical mean and variance of the estimator is derived and analyzed and comparison with the MVAM estimation is made. Mathematical simulations for both the linear array case and planar array case are formulated in Section 5. Finally, concluding remarks are given in Section 6.

## 2. Data Model

Consider a linear array consisting of *m* isotropic elements with spacing *d*. Assume a target signal impinges on the array with amplitude *b* and phase *ξ* and incidental angle $\theta_{0}$ which is the physical angle with respect to the line of the array. Besides the target, assume there are *p* strong sources of interference emitting jamming noise from different incidental angles $\left\{\theta_{i}:i=1,2\cdots p\right\}$ and with amplitudes $\left\{x_{i}:i=1,2\cdots p\right\}$ and phases $\left\{\xi_{i}:i=1,2\cdots p\right\}$ respectively. Let

(1) $$a(\theta )=\left[1\;e^{j\frac{2\pi d}{\lambda }\cos\left(\theta \right)}\;\cdots \;e^{j\frac{2\pi (m-1)d}{\lambda }\cos\left(\theta \right)}\right]$$

denote the array response to a plane wave of unit amplitude arriving at an incidental angle *θ*, *j* is the unit of imaginary numbers and *λ* is the wavelength of the transmitted signal in radar. Then, the array’s output at the *k*th moment can be written as

(2) $$y\left(k\right)=b\left(k\right)e^{j\xi (k)}a\left(\theta_{0}\right)+\sum_{i=1}^{p}x_{i}(k)e^{j\xi_{i}\left(k\right)}a(\theta_{i})+n\left(k\right),$$

where n(k) is the time sample at the *k*-th moment from white Gaussian noise. With the array output, the monopulse technique requires forming a sum beam and a difference beam by cutting the array into its left half sub-array and right half sub-array. The direction of the mainlobe of the sum beam is determined by the radar look direction *θ*. However, in the adaptive monopulse radar, a slight deviation may exist due to the presence of interference [12]. If the target is located right at the radar look direction, the sum beam output will be maximum while the difference beam will be zero. If the target has a small offset angle from the look direction $\theta_{0}-\theta$, the ratio of the sum to difference beam, namely the monopulse ratio is employed to estimate this angle. The monopulse ratio is approximately independent of the target amplitude and has a good linear shape over the offset angle. For amplitude monopulse, one can easily show the following basic relation by Taylor expansion [27] with a fixed squint angle *δ* and antenna amplitude pattern *h*

(3) $$\frac{h\left(\theta_{0}-\theta -\delta \right)-h\left(\theta_{0}-\theta +\delta \right)}{h\left(\theta_{0}-\theta -\delta \right)+h\left(\theta_{0}-\theta +\delta \right)}\approx -\frac{h^{\prime }\left(\delta \right)}{h(\delta )}\cdot \left(\theta_{0}-\theta \right),$$

where ${\left(\right)}^{\prime }$ is the first order derivative. However, the sum beam is incoherently formed in this way as we can see from (3), which leads to loss of gain. It is for this reason that we design our algorithm based on the phase monopulse technique. By ignoring noise and interference, the outputs of two sub-arrays have the form $be^{j\xi }h\left(\theta_{0}-\theta \right)e^{j\frac{2\pi \bar{d}}{\lambda }\left(\cos\left(\theta_{0}\right)-\cos\left(\theta \right)\right)}$ and $be^{j\xi }h\left(\theta_{0}-\theta \right)e^{-j\frac{2\pi \bar{d}}{\lambda }\left(\cos\left(\theta_{0}\right)-\cos\left(\theta \right)\right)}$ for phase monopulse where *h* denotes the common sub-array pattern and $\bar{d}$ denotes the distance from the phase center of each sub-array to the center of the whole array. Therefore, one can easily get the monopulse ratio $r=j\cdot \tan2\pi \frac{\bar{d}}{\lambda }\left(\cos\left(\theta_{0}\right)-\cos\left(\theta \right)\right)$, which is again a linear function in the neighborhood of the look direction.

In the presence of the interference and noise, the aim is to suppress both the interference and noise as well as extracting the angle to the target. To fulfil this task, appropriate sum and difference beam weights needs to be found that are as orthogonal to the interference subspace as possible while maintaining linearity within the neighborhood of the look direction. Denoting such sum beam weights as $w_{sum}$ and difference beam weights as $w_{diff}$, we have

(4) ImwdiffHy(k)wsumHy(k)≈Γ(θ0−θ),

where Im· is the imaginary part of a complex variable, ${\left(\cdot \right)}^{H}$ is the conjugate transpose and Γ is the slope of monopulse ratio. Consequently, the offset angle of target can be easily estimated using (4).

For a planar array, the array manifold becomes

(5) $$a(\theta ,\varphi )=\left[\begin{array}{ccc} 1 & \cdots & e^{j\frac{2\pi }{\lambda }(m-1)d\cos\theta +j\frac{2\pi }{\lambda }(n-1)d\cos\varphi } \end{array}\right],$$

where *θ* and *φ* are the incident angles w.r.t. the *X* and *Y* axis and named azimuth and elevation respectively, *m* and *n* are the number of elements along the corresponding dimension. In this case, the estimates of azimuth and elevation are correlated with each other. In fact, by using Taylor expansion, it can be seen that the monopulse ratio is correlated with two partial derivatives w.r.t. azimuth or elevation [18]. Thus, estimation of either azimuth or elevation would require knowledge of the other, which makes the problem harder to approach. However, with difference beam weights appropriately chosen, we can decouple this two-dimensional problem into two independent one-dimensional monopulse estimations and use (4) to solve them separately.

## 3. The Proposed Constrained Algorithm

### 3.1. Derivation of the Algorithm Used in a Linear Array

In this section, the proposed algorithm is introduced and the derivation of the constrained difference beam weights given. The procedure commences by first forming the adaptive sum beam weights using a minimum variance distortionless response beamformer (MVDR) [8], e.g.,

(6) $$w_{sum}=\frac{R^{-1}a\left(\theta \right)}{a^{H}\left(\theta \right)R^{-1}a\left(\theta \right)},$$

where R is the covariance of the interference plus noise and can be estimated by the ML estimator

(7) $$R=\frac{1}{N}\sum_{k=1}^{N}\tilde{y}\left(k\right){\tilde{y}}^{H}\left(k\right),$$

where *N* is number of snapshots used for estimation and $\tilde{y}(k)$ is the sample of interference plus noise, which can be obtained using the interference covariance matrix reconstruction methods [28,29]. The diagonal loading technique can also be applied when forming the sum beam in case mismatch exists between the radar look direction and the target’s true direction.

The calculation of difference in beam weights is dependent on the sum beam weights obtained in (6). The difference beam weights are not only required to obtain sufficient suppression of interference and noise, but also to keep the approximation in (4) valid. Therefore, we formulate the following problem to derive the difference beam weight:

(8) minwdiffwdiffHRwdiff,s.t.Im(wdiffHC)=g

where matrix C is

(9) $$C=\left[\frac{a(\theta +\Delta \theta )}{w_{sum}^{H}a\left(\theta +\Delta \theta \right)}\;\frac{a(\theta )}{w_{sum}^{H}a\left(\theta \right)}\right],$$

and row vector g is

(10) $$g=\left[\Gamma \Delta \theta \;0\right],$$

where Δθ is a small user-determined parameter whose value influences the performance of the proposed algorithm. The choice of Δθ will be discussed shortly.

Using the Lagrange Multiplier method to approach the constrained problem in (8) gives

(11) minwdiff,pf=wdiffHRwdiff+Im(wdiffHC)p),

where p is a real column vector to be determined. To solve (11), a complex gradient is introduced as follows [30]:

(12) ∇wdiff*(f)=∂f∂Re(wdiff)+j∂f∂Im(wdiff)/2

where Re(·) is the real part of a complex variable. With (11) and (12), we have

(13) $$\begin{array}{c} \nabla_{w_{diff}^{*}}(f)=Rw_{diff}-\frac{j}{2}\mathrm{Cp}, \end{array}$$

where we have used the fact that R is Hermitian. To find a minimum point for *f*, set the gradient to zero, then we have

(14) $$w_{diff}=\frac{j}{2}R^{-1}\mathrm{Cp}$$

Substituting (14) into the constraint of (8) gives

(15) p=−2ReCHR−1C−TgT,

where ${\left(\cdot \right)}^{T}$ is the transpose of a matrix and ${\left(\cdot \right)}^{-T}$ is the transpose of the inverse matrix. Combining (14) and (15) together, we give the solution to (8) as follows:

(16) wdiff=−jR−1CReCHR−1C−TgT

From (4) and (16), it can be seen that the value of Γ has no effect on the estimate of $\theta_{0}$. Therefore, it can be any real number other than 0. For simplicity, we make Γ=1 for the rest of the paper.

After getting the sum beam weight from (6) and the difference beam weight from (16), the monopulse ratio can be easily calculated and therefore the target angle.

The optimization problem in (8) seeks the solution that minimizes the power of interference plus noise under two hard constraints. Since the interference and noise are the source of variance of monopulse estimation, minimizing their power is equal to minimizing the variance of monopulse estimation. The hard constraints clamp the values of monopulse ratio at two close positions, forcing the imaginary part of the monopulse ratio to satisfy (4) within the neighborhood of the radar look direction *θ*. One of the constraints guarantees that the difference beam has a null at *θ*, which eliminates estimation bias. The other ensures the approximate linearity within [θ−Δθ,θ+Δθ], if Δθ is small. The reason for this can be explained as follows. Suppose the target angle being $\theta_{0}=\theta +\tilde{\lambda }\Delta \theta ,\left|\tilde{\lambda }\right|\leq 1$ where || means the absolute value or the modulus. By using Taylor expansion for the array manifold and omitting infinitesimal of higher order, we have

(17) $$a\left(\theta +\tilde{\lambda }\Delta \theta \right)\approx a\left(\theta \right)+\tilde{\lambda }\left(a\left(\theta +\Delta \theta \right)-a\left(\theta \right)\right),$$

After beamforming, the output SNIR of both sum and difference beam should be high enough, thus the monopulse ratio can be written as

(18) Im(r)≈ImwdiffH(a(θ)+λ˜(a(θ+Δθ)−a(θ)))wsumH(a(θ)+λ˜(a(θ+Δθ)−a(θ))),

Considering Δθ is small and the numerator is much smaller than the denominator in (18), we have the following approximation:

(19) $$w_{sum}^{H}a\left(\theta \right)\approx w_{sum}^{H}a\left(\theta +\Delta \theta \right)$$

when calculating the imaginary part of monopulse ratio. From the constraints, we have

(20) ImwdiffHa(θ)wsumHa(θ)=0,ImwdiffHa(θ+Δθ)wsumHa(θ+Δθ)=Δθ.

Combining (18) and (20), one can easily get

(21) ImwdiffHywsumHy≈λ˜Δθ,λ˜≤1.

which is equal to (4) within [θ−Δθθ+Δθ].

From the above analysis, it is clear that the constraints in (20) are able to clamp the monopulse ratio. For convenience, we have assumed that the angle of target lies in [θ−Δθ,θ+Δθ]. However, the angle of target does not have to be confined in this range. As long as $\tilde{\lambda }\Delta \theta$ is small enough to keep (17) and (19) valid, a reasonable estimation of the target’s offset angle can still be obtained by (4). However, with the target moving further away from the radar look direction, the approximation error (non-linearity error) will grow, thus the performance of estimation can be expected to deteriorate. Therefore, as long as Δθ is small, the range of angle estimation does not rely on Δθ.

Indeed, the choice of Δθ should be able to keep (17) and (19) valid which demands that θ+Δθ lies within the neighborhood of the look direction. Denote the range of this neighborhood as *ε*. In practice, *ε* is usually considered to be the 3 dB beamwidth of the sum beam. With the target also lying within the neighborhood of the look direction, the approximation error is usually negligible compared to the error incurred by noise and interference. Therefore, the choice of Δθ should be made to minimize the power of noise and interference

(22) $$\Delta \theta =\min_{\Delta \theta }\;\left(w_{diff}^{H}Rw_{diff}\right),s.t.\Delta \theta \in \left[\theta -\varepsilon /2,\;\theta +\varepsilon /2\right]\backslash \left\{0\right\}.$$

With (16), we have

(23) wdiffHRwdiff=gReCHR−1C−1CHR−1CReCHR−1C−1gH.

Combining (22) and (23), we can get

(24) Δθ=minΔθF=Δθ2det(Re(CHR−1C)),s.t.Δθ∈[θ−ε/2θ+ε/2]\{0}.

which is a non-linear optimization problem. However, using Taylor expansion for the array manifold and omitting infinitesimal of higher order, we can rewrite (24) as

(25) $$F\approx \frac{\mu^{2}}{{\left|\mu +\eta \Delta \theta \right|}^{2}\left(\mu \nu -{\left|\eta \right|}^{2}\right)},$$

where $\mu =a^{H}\left(\theta \right)R^{-1}a\left(\theta \right)$, $\nu =a^{\prime H}\left(\theta \right)R^{-1}a^{\prime }\left(\theta \right)$, $\eta =a^{\prime H}\left(\theta \right)R^{-1}a\left(\theta \right)$. The denominator of (25) is a quadratic function of Δθ. Its axis of symmetry is at Δθ=−Re(η)μη2. From Cauchy–Schwarz inequality, $\mu \nu \geq {\left|\eta \right|}^{2}$. Therefore, the choice of Δθ depends on the sign of Re(η):

(26) Δθ=ε2ifRe(η)>0−ε2ifRe(η)<0

It should be noted that the performance improvement brought by optimizing Δθ depends on the value of −Re(η)μη2. If Re(η)μη2≫ε/2, the improvement will be more prominent. However, if Re(η)μη2→0, this improvement would be less obvious.

Function *F* is not continuous at Δθ=0, but we can make it continuous by assigning value to it. Denote

(27) F(0)=limΔθ→0Δθ2detReCHR−1C≈1μν−η2.

In fact, as Δθ approaches zero from positive axis, we have

(28) limΔθ→0ImwdiffHa(θ+Δθ)wsumHa(θ+Δθ)−ImwdiffHa(θ)wsumHa(θ)Δθ=1.

The constraints in (20) are equal to the demanding derivative of the imaginary part of the monopulse ratio at the radar look direction, to be one with a bias of zero. This case, as we will show in next section, is approximately equal to the MVAM.

Since the constraints are enough to clamp the monopulse ratio, adding extra constraint has very little effect on improving linearity, but it might incur extra loss of DOF. For this reason, the constraint at θ−Δθ as in [24] has little effect on improving linearity but could result in more loss of DOF. Furthermore, the constraint used in [24] could not be applied to a complex signal. Therefore, the proposed algorithm is superior to the algorithm in [24].

### 3.2. Extension to Planar Array Application

In a typical planar phased array, the sum and difference beamforming is usually done at the sub-array level using the digital output of the sub-arrays to reduce the dimension of computation. When adaptive beamforming is applied, a serious problem can arise in that the monopulse ratio curve of one dimension may be dependent on the value of the other. This problem can be severe enough to make the monopulse technique fail. Consider a planar array placed in the *X*–*Y* plane of a Cartesian system with the center of array overlapping with the origin. The array manifold is shown in (5).

Unlike the linear array case, to estimate either azimuth or elevation angle, additional constraint is required to make the estimation of one angle independent on the value of the other. Therefore, the constrained problem that generates difference beam weights takes the form:

(29) minwazwazHRwaz,minwelwelHRwels.t.ImΩHC2=G2,Ω=wazwel

where $w_{az}$ and $w_{el}$ are the azimuth and elevation difference beam adaptive weights and

(30) $$\begin{array}{c} C_{2}=\left(\begin{array}{ccc} \frac{a(\theta ,\varphi )}{w_{sum}^{H}a\left(\theta ,\varphi \right)} & \frac{a(\theta +\Delta \theta ,\varphi )}{w_{sum}^{H}a\left(\theta +\Delta \theta ,\varphi \right)} & \frac{a(\theta ,\varphi +\Delta \varphi )}{w_{sum}^{H}a\left(\theta ,\varphi +\Delta \varphi \right)} \end{array}\right),\\ G_{2}=\left(\begin{array}{ccc} 0 & \Delta \theta & 0\\ 0 & 0 & \Delta \varphi \end{array}\right). \end{array}$$

Using the Lagrange Multiplier method gives

(31) Ω=−jR−1C2ReC2HR−1C2−TG2T.

The reason for the constraints keeping linearity within the neighborhood of the radar look direction can be demonstrated in a way similar to the case of linear arrays. Let (θ,φ) denote the radar look direction. The area of the neighborhood is composed by azimuth ranging from $\left(\theta -\varepsilon /2\right)$ to $\left(\theta +\varepsilon /2\right)$ and elevation ranging from $\left(\varphi -\delta /2\right)$ to $\left(\varphi +\delta /2\right)$. Practically, this area is also determined by the 3 dB width of the mainlobe. Assume the following approximation holds within the neighborhood of the radar look direction:

(32) $$\begin{array}{cc} a\left(\tilde{\theta },\tilde{\varphi }\right) & =\left(1-\lambda_{\theta }\right)\left(1-\lambda_{\varphi }\right)a\left(\theta ,\varphi \right)\\ & +\left(1-\lambda_{\theta }\right)\lambda_{\varphi }a\left(\theta ,\varphi +\Delta \varphi \right)\\ & +\lambda_{\theta }\left(1-\lambda_{\varphi }\right)a\left(\theta +\Delta \theta ,\varphi \right) \end{array}$$

(33) $$w_{sum}^{H}a\left(\theta ,\varphi \right)\approx w_{sum}^{H}a\left(\tilde{\theta },\tilde{\varphi }\right)$$

where $\tilde{\theta }=\theta +\lambda_{\theta }\Delta \theta \in \left[\begin{array}{cc} \theta -\varepsilon /2, & \theta +\varepsilon /2 \end{array}\right]$ and $\tilde{\varphi }=\varphi +\lambda_{\varphi }\Delta \varphi \in \left[\begin{array}{cc} \varphi -\delta /2, & \varphi +\delta /2 \end{array}\right]$. Then, with the constraints in (29), one can easily have

(34) ImΩHaθ˜,φ˜wsumHaθ˜,φ˜≈λθΔθλφΔφ

Optimization of parameters Δθ and Δφ requires solving the following non-linear problem:

(35) $$\begin{array}{c} \min_{\Delta \theta ,\Delta \varphi }\;\left(tr\left(K\Omega^{H}R\Omega \right)\right),K=\left(\begin{array}{cc} k_{az} & 0\\ 0 & k_{el} \end{array}\right)\\ s.t.\Delta \theta \in \left[\begin{array}{cc} -\varepsilon /2, & \varepsilon /2 \end{array}\right]\backslash \left\{0\right\},\Delta \varphi \in \left[\begin{array}{cc} -\delta /2, & \delta /2 \end{array}\right]\backslash \left\{0\right\} \end{array}$$

where tr(·) is the trace of a matrix, K is a weighting matrix that weights the variance of azimuth and elevation difference beam outputs. Unfortunately, there is no easy way to solve this problem as in the linear array case. Thus numerical methods such as the Nelder–Mead method are suggested for this problem. However, as long as the constrained directions θ+Δθ and φ+Δφ lie within the neighborhood of the radar look direction, the proposed algorithm with optimized Δθ and Δφ can always achieve a better performance than the previous method, e.g., the MVAM.

### 3.3. Summary and Computational Complexity of the Proposed Algorithm

We conclude the procedure of the proposed monopulse technique and discuss the computational complexity as follows:

1. calculate the adaptive sum beam weights using (6). In this step, calculation of the sample matrix inversion (SMI) is the most expensive. Fortunately, we can use the recursive matrix inversion formula for the one rank updated sample covariance matrix [31] which can reduce the computational complexity to the level of $O\left(l^{2}\right)$ where *l* is the dimension of the array manifold. The other matrix multiplication is also in the order of $O\left(l^{2}\right)$ [32,33]. Therefore, the total computational complexity in this step is $O\left(l^{2}\right)$.
2. Determine the parameter Δθ or the parameter pair (Δθ,Δφ) as follows: in the linear array case, use (26) to determine Δθ, while in the planar array case, solve (35) for (Δθ,Δφ) or directly make Δθ and Δφ close to zero to approximate the performance of the MVAM if seeking to reduce computational cost. In this step, determining the parameter Δθ according to (26) or directly choosing a small value close to zero does not require any computation. However, in the planar array case, optimization of the parameters requires recursive iteration to solve (35), thus it is not suggested for real-time applications.
3. Use (16) for linear array applications or (31) for planar array applications to calculate the constrained difference beam weights. The sample matrix inversion is already calculated in the first step and the other matrix inversion in (16) and (31) is usually negligible because l≫3 in practice. Therefore, the total computational complexity in this step is in the order of $O\left(l^{2}\right)$.
4. Perform beamforming with the beam weights calculated in the previous steps and then calculate the monopulse ratio along with the angle estimates. This last step has the computational complexity of $O\left(2l\right)$.

In general, the total computational complexity of the above procedure is $O\left(l^{2}\right)$ which is about the same as the MVAM. In practice, such computational cost is usually affordable for modern radar systems.

It is worth noting that before forming the monopulse ratio, any linear processing, such as coherent integration, can be applied to both beam outputs to improve SNIR as the processing effect on the target signal is identical in both beam outputs.

## 4. Performance Analysis of the Proposed Monopulse Estimator

### 4.1. Mean and Variance of the Proposed Estimator

In this section, the theoretical performance of the proposed estimator is analyzed. The derivation follows the ideas developed in [17,34,35] using conditional distributions. All the beam weights are considered to be given quantities. Let $d_{x}$ and $d_{y}$ denote the output of the azimuth difference beam and elevation difference beam respectively. *s* is the output of the sum beam. The beam outputs ${\left(\begin{array}{ccc} d_{x} & d_{y} & s \end{array}\right)}^{T}$ are assumed to be circularly-symmetric complex Gaussian distributed with mean u and covariance matrix Q. The mean vector and covariance matrix can be partitioned as

(36) $$u=\left(\begin{array}{c} u_{d}\\ u_{s} \end{array}\right),\;Q=\left(\begin{array}{cc} Q_{d} & q_{ds}\\ q_{ds}^{H} & q_{s} \end{array}\right),$$

where $u_{d}$ is the mean of $d={\left(\begin{array}{cc} d_{x} & d_{y} \end{array}\right)}^{T}$, $Q_{d}$ is its covariance, $q_{ds}=E\left(\left(d-u_{d}\right){\left(s-u_{s}\right)}^{H}\right)$ is the covariance with *s* and E(·) means the expectation value. Here, we only consider the case of the deterministic target. Therefore, we have

(37) $$u_{d}=be^{j\xi }\Omega^{H}a\left(\theta_{0},\varphi_{0}\right),\;u_{s}=be^{j\xi }w_{sum}^{H}a\left(\theta_{0},\varphi_{0}\right);$$

where $\left(\theta_{0},\varphi_{0}\right)$ is the direction of the target and

(38) $$Q_{d}=\Omega^{H}R\Omega ,\;q_{s}=w_{sum}^{H}Rw_{sum},\;q_{ds}=\Omega^{H}Rw_{sum}.$$

The conditional distribution of d conditioned on *s* is also complex Gaussian with mean $u_{d|s}=u_{d}+q_{ds}q_{s}^{-1}\left(s-u_{s}\right)$ and covariance $Q_{d|s}=Q_{d}-q_{ds}q_{s}^{-1}q_{ds}^{H}$. Rewrite the imaginary part of the monopulse ratio as

(39) r=Imds*s2.

Then, the conditional distribution of r conditioned on *s* is Gaussian with mean Imqdsqs−1+ud−qdsqs−1uss−1 and covariance ReQd|s2s2. Let $u_{az|s}$ and $q_{az|s}$ denote the mean and variance of the azimuth estimator conditioned on *s* respectively. With (37) and (38), they can be written as

(40) uaz|s=Imβ+wazHaθ0,φ0−βwsumHaθ0,φ0bejξs−1,qaz|s=wazHRwaz−wazHRwsum2wsumHRwsum2s2−1,β=wazHRwsumwsumHRwsum.

The performance of the estimator depends on the sum beam weights. In the presence of interference, the MVDR beamformer, as in (6), is usually a practical method for calculating sum beam weights and the proposed monopulse technique is based on it.

Using (6) and the constraint Imβ=0, we can get

(41) uaz|s=ImwazHaθ0,φ0−wazHaθ,φwsumHaθ0,φ0bejξs−1,qaz|s=wazHRwaz−wazHaθ,φ2aHθ,φR−1aθ,φ2s2−1.

By integrating over the distribution of *s*, the mean and covariance of r can then be obtained. The sum beam output power ${\left|s\right|}^{2}$ cannot be too low, otherwise the monopulse estimation would fail. Therefore, a threshold *α* is set for the lower bound of the integration and the mean and variance derived in the following is still conditioned on ${\left|s\right|}^{2}>\alpha$ [17]. This threshold is relevant with the scenario where the radar system is working, including SNIR, false alarm rate, etc. Using the results from [17], the mean of the azimuth estimator $u_{az}$ is given by

(42) uaz=ImwazHaθ0,φ0wsumHaθ0,φ01−a1pd

where

(43) $$\begin{array}{c} a_{1}=e^{-\left(\alpha +{\left|u_{s}\right|}^{2}\right)/q_{s}}\cdot I_{0}\left(2\sqrt{\alpha }\left|u_{s}\right|/q_{s}\right),\\ p_{d}=\frac{1}{q_{s}}\int_{\alpha }^{\infty }e^{-\left(t+{\left|u_{s}\right|}^{2}\right)/q_{s}}\cdot I_{0}\left(2\sqrt{t}\left|u_{s}\right|/q_{s}\right)\;dt \end{array}$$

where $I_{0}$ denotes the modified Bessel function of order zero. In the derivation of (42), we have used the constraint Imβ=0. The mean of the elevation estimator can be derived analogously:

(44) uel=ImwelHaθ0,φ0wsumHaθ0,φ01−a1pd

As *α* approaches zero, $a_{1}$ is approaching a small value determined by the SNR of the sum beam and $p_{d}$ is approaching unity. Thus, the proposed estimator is approximately unbiased.

With the results from [17], the variance of the azimuth and elevation estimator is

(45) $$\begin{array}{cc} q_{az} & =\frac{1}{2}\left(w_{az}^{H}Rw_{az}-\frac{{\left|w_{az}^{H}a\left(\theta ,\varphi \right)\right|}^{2}}{a^{H}\left(\theta ,\varphi \right)R^{-1}a\left(\theta ,\varphi \right)}\right)\frac{a_{2}}{p_{d}}\\ & =\frac{1}{2}\left(w_{az}^{H}R^{1/2}P_{null}R^{1/2}w_{az}\right)\frac{a_{2}}{p_{d}},\\ q_{el} & =\frac{1}{2}\left(w_{el}^{H}Rw_{el}-\frac{{\left|w_{el}^{H}a\left(\theta ,\varphi \right)\right|}^{2}}{a^{H}\left(\theta ,\varphi \right)R^{-1}a\left(\theta ,\varphi \right)}\right)\frac{a_{2}}{p_{d}}\\ & =\frac{1}{2}\left(w_{el}^{H}R^{1/2}P_{null}R^{1/2}w_{el}\right)\frac{a_{2}}{p_{d}}, \end{array}$$

where $P_{null}$ is the projection matrix to the null space of $R^{-1/2}a\left(\theta ,\varphi \right)$ and

(46) $$a_{2}=\int_{\alpha }^{\infty }\frac{1}{q_{s}}e^{-\left(t+{\left|u_{s}\right|}^{2}\right)/q_{s}}\cdot I_{0}\left(2\sqrt{t}\left|u_{s}\right|/q_{s}\right)t^{-1}dt.$$

With (31), we can rewrite ReΩHaθ,φ as

(47) ReΩHaθ,φ=G2ReC2HR−1C2−1ImC2HR−1aθ,φ.

With the constraints in (29), one can easily get ImC2HR−1aθ,φ=0. Therefore, we have

(48) $$\begin{array}{c} q_{az}=\frac{1}{2}\left(w_{az}^{H}Rw_{az}\right)\frac{a_{2}}{p_{d}},\\ q_{el}=\frac{1}{2}\left(w_{el}^{H}Rw_{el}\right)\frac{a_{2}}{p_{d}}. \end{array}$$

As we can see from (48), $\frac{a_{2}}{p_{d}}$ is determined by the SNR of the sum beam output and the threshold *α*, the other term is the variance of the difference beam output. Therefore, given the sum beam weights and *α*, the variance of the estimator only depends on the variance of the difference beam output which hence explains (22) and (35) for the optimization of parameter Δθ.

### 4.2. Comparison with MVAM

In the MVAM [18], the adaptive difference beam weights were derived under different objective function and different constraints from (29). However, as will be proved in the following, the adaptive difference beam weights calculated by the MVAM are equivalent with that of the proposed algorithm in a special case where Δθ→0. Therefore, the proposed algorithm can outperform the MVAM with the parameter Δθ optimized using (26) or in the planar array case, using numerical methods. Let $\tilde{\Omega }=\left(\begin{array}{cc} {\tilde{w}}_{az} & {\tilde{w}}_{el} \end{array}\right)$ denote the adaptive difference beam weights given by the MVAM. These weights are calculated from [18]:

(49) minw˜azw˜azHR˜w˜az,minw˜elw˜elHR˜w˜els.t.ReC˜HΩ˜=I2

where $I_{2}$ is the unity matrix of rank 2 and

(50) $$\begin{array}{cc} \tilde{R} & =R-\frac{a\left(\theta ,\varphi \right)w_{sum}^{H}}{w_{sum}^{H}a\left(\theta ,\varphi \right)}R-R\frac{w_{sum}a{\left(\theta ,\varphi \right)}^{H}}{a^{H}\left(\theta ,\varphi \right)w_{sum}}\\ & \;\;+\frac{a\left(\theta ,\varphi \right)a^{H}\left(\theta ,\varphi \right)}{{\left|w_{sum}^{H}a\left(\theta ,\varphi \right)\right|}^{2}}w_{sum}^{H}Rw_{sum}\\ \tilde{C} & =\left(\begin{array}{cc} c_{az} & c_{el} \end{array}\right),\\ c_{az} & =\frac{\left(w_{sum}^{H}a\left(\theta ,\varphi \right)\right)d_{\theta }-\left(w_{sum}^{H}d_{\theta }\right)a\left(\theta ,\varphi \right)}{{\left|w_{sum}^{H}a\left(\theta ,\varphi \right)\right|}^{2}},\\ c_{el} & =\frac{\left(w_{sum}^{H}a\left(\theta ,\varphi \right)\right)d_{\varphi }-\left(w_{sum}^{H}d_{\varphi }\right)a\left(\theta ,\varphi \right)}{{\left|w_{sum}^{H}a\left(\theta ,\varphi \right)\right|}^{2}}, \end{array}$$

where $d_{\theta }$ and $d_{\varphi }$ are respectively the partial derivatives of $a\left(\theta ,\varphi \right)$ w.r.t. *θ* and *φ* at the look direction. The singularity of $\tilde{R}$ prevents solving (49) directly with the Lagrange method. In [18], this problem was solved by adding an additional constraint ${\tilde{\Omega }}^{H}w_{sum}=0$.

Because $R^{-1}=MPM^{H}+\frac{1}{\sigma^{2}}I$ where M is the interference eigenvector matrix and $\sigma^{2}$ is the power of the noise, it follows that

(51) $${\tilde{\Omega }}^{H}w_{sum}=\frac{\left({\tilde{\Omega }}^{H}\mathrm{MP}M^{H}a\left(\theta ,\varphi \right)+\frac{1}{\sigma^{2}}{\tilde{\Omega }}^{H}a\left(\theta ,\varphi \right)\right)}{a^{H}\left(\theta ,\varphi \right)R^{-1}a\left(\theta ,\varphi \right)}.$$

Because the power of interference is assumed to be much stronger than the noise, the maximum eigenvalue of P is much smaller than $\frac{1}{\sigma^{2}}$ and the difference beam weights should be orthogonal to the sub-space of interference. Therefore, we have

(52) $${\tilde{\Omega }}^{H}w_{sum}\approx \frac{1}{a^{H}\left(\theta ,\varphi \right)R^{-1}a\left(\theta ,\varphi \right)}\left(\frac{1}{\sigma^{2}}{\tilde{\Omega }}^{H}a\left(\theta ,\varphi \right)\right)$$

and further the constraint ${\tilde{\Omega }}^{H}w_{sum}=0$ approximately equals to ${\tilde{\Omega }}^{H}a\left(\theta ,\varphi \right)=0$. Thus, with the additional constraint ${\tilde{\Omega }}^{H}a\left(\theta ,\varphi \right)=0$ added to (49), the solution of the MVAM is given by:

(53) Ω˜=R¯−1C¯ReC¯HR¯−1C¯−1100100,R¯=R˜+a3aθ,φaHθ,φ,C¯=C˜aθ,φ

where $a_{3}$ is a non-zero loading factor that makes $\bar{R}$ invertible. The effect of the loading matrix is cancelled by the additional constraint.

Let $a_{3}=1/a^{H}\left(\theta ,\varphi \right)R^{-1}a\left(\theta ,\varphi \right)$, then the weight solution becomes

(54) Ω˜=R−1C¯ReC¯HR−1C¯−1100100

Let us rewrite $\bar{C}$ as follows:

(55) $$\begin{array}{c} \bar{C}=\lim_{\begin{array}{c} \Delta \theta \to 0\\ \Delta \varphi \to 0 \end{array}}\;\left(\Pi \right),\;\Pi =\left(\begin{array}{ccc} z_{1} & z_{2} & z_{3} \end{array}\right),\\ z_{1}=\frac{1}{\Delta \theta }\left(\frac{a\left(\theta +\Delta \theta ,\varphi \right)}{w_{sum}^{H}a\left(\theta +\Delta \theta ,\varphi \right)}-\frac{a\left(\theta ,\varphi \right)}{w_{sum}^{H}a\left(\theta ,\varphi \right)}\right),\\ z_{2}=\frac{1}{\Delta \varphi }\left(\frac{a\left(\theta ,\varphi +\Delta \varphi \right)}{w_{sum}^{H}a\left(\theta ,\varphi +\Delta \varphi \right)}-\frac{a\left(\theta ,\varphi \right)}{w_{sum}^{H}a\left(\theta ,\varphi \right)}\right),\\ z_{3}=\frac{a\left(\theta ,\varphi \right)}{w_{sum}^{H}a\left(\theta ,\varphi \right)}. \end{array}$$

Combining (54) and (55), we have

(56) Ω˜=limΔθ→0Δφ→0R−1ΠReΠHR−1Π−1100100=limΔθ→0Δφ→0jΩ.

where Ω is calculated by (31). It can be seen from (56) that the MVAM corresponds to a special case of the proposed algorithm where Δθ→0 and Δφ→0. In the case of linear array, one can prove that the variance of estimation using the MVAM is ${\left(\mu \nu -{\left|\eta \right|}^{2}\right)}^{-1}$ which is (27).

Denote $G\left(\Delta \theta ,\Delta \varphi \right)=tr\left(K\Omega^{H}R\Omega \right)$ and define G(0,0) by its limit value. Then, function *G* is continuous almost everywhere within the domain $\Delta \theta \in \left[\begin{array}{cc} -\varepsilon /2, & \varepsilon /2 \end{array}\right],\Delta \varphi \in \left[\begin{array}{cc} -\delta /2, & \delta /2 \end{array}\right]$. Therefore, by optimizing the choice of Δθ and Δφ, the proposed algorithm could achieve better performance than the MVAM or Nickel’s corrected adaptive monopulse [18] which gives the same performance as the MVAM. In the case of linear array, the strategy for choosing the parameter is given by (26).

Furthermore, the proposed algorithm does not require knowledge of the derivatives of array response. Thus, it is more robust than the MVAM in the case where errors about the array response exist.

## 5. Numerical Examples and Applications

In order to examine the performance of the proposed monopulse algorithm, mathematical simulations are conducted using an echo simulation software programmed in MATLAB. Comparison is made between the proposed algorithm, traditional constrained method [24] and the MVAM. The simulation is composed of two parts. In the first part, different methods are compared using a uniform linear array, while in the second they are applied to a rectangular planar array for comparison.

### 5.1. Simulation in Linear Array

**Figure 1:**
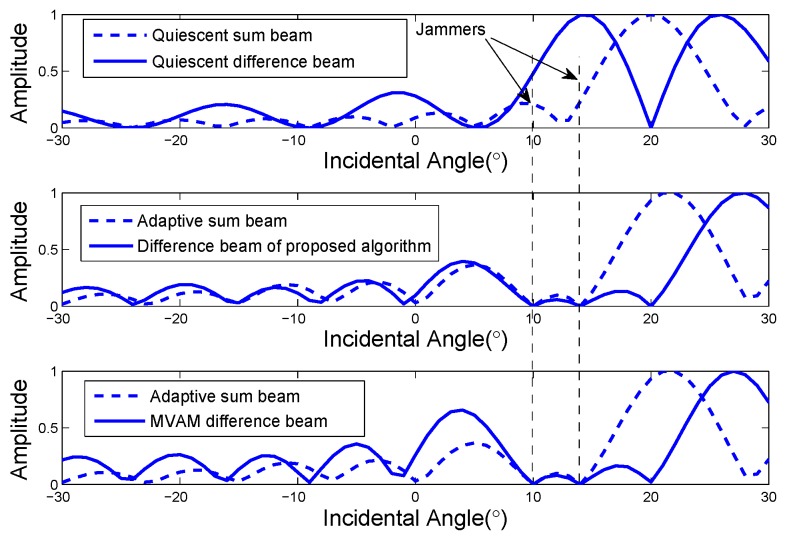
The beam pattern of the proposed algorithm.

Consider a linear array with 16 elements equally spaced by half of the wavelength. The desired radar look direction is 20${}^{\circ }$ away from the boresight. A deterministic target is located at the direction 20.8${}^{\circ }$ off the boresight with the power of 10 dB relative to internal noise. Two noise jammers with the power of 30 dB relative to internal noise are incident from 10${}^{\circ }$ and 14${}^{\circ }$ off the boresight respectively. The sum beam is formed adaptively using (6). The beam patterns using the proposed algorithm and the MVAM are plotted in Figure 1. As a reference, the quiescent beam patterns are also plotted. For the proposed algorithm, Δθ is set to be 4${}^{\circ }$ (half of the 3 dB beamwidth), because Reη>0. It can be seen from Figure 1 that the mainlobe of the sum beam pattern is slightly nudged away from the desired look direction due to the strong jammers. Two clear nulls can be observed at the directions of jammers for both of the methods, providing jammer rejection. However, the proposed algorithm places another null at the radar look direction because of the constraint while the MVAM only tends to have a null at the look direction [18].

**Figure 2:**
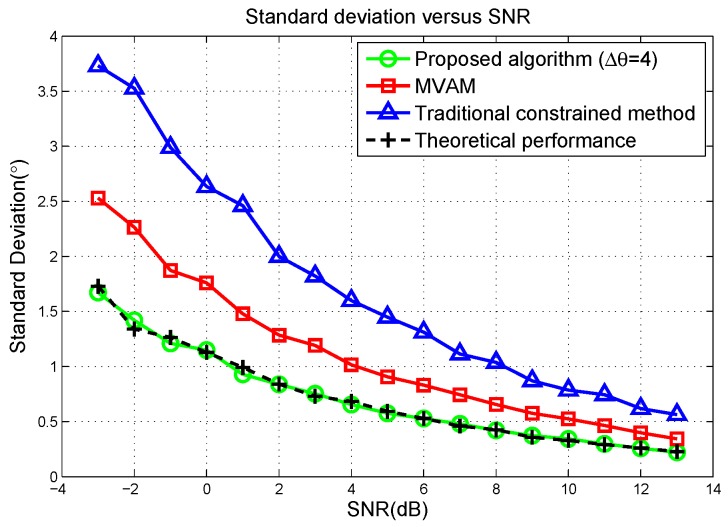
Mean of the estimation error versus the signal-to-noise ratio (SNR) in scenario 1 of the linear array simulation.

**Figure 3:**
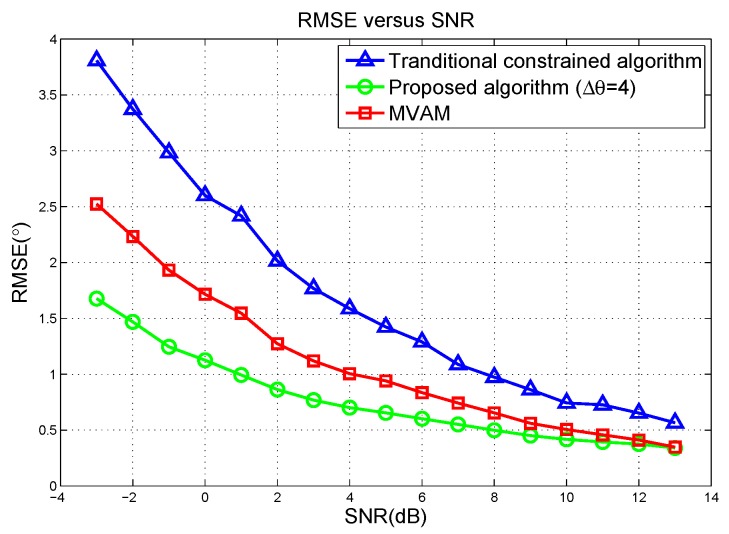
Standard deviation versus the SNR in scenario 1 of linear array simulation.

In Figure 2, the standard deviations of the proposed algorithm, the MVAM and the traditional constrained method are plotted with signal to noise ratio (SNR) changing from −3 dB to 13 dB and Root-Mean-Square Errors (RMSE) of these three methods are plotted in Figure 3. The covariance matrix R is estimated from 100 snapshots. For the traditional constrained method, the constrained points are respectively −4${}^{\circ }$, 0${}^{\circ }$ and 4${}^{\circ }$ away from the radar look direction. It can be seen that the proposed algorithm outperforms both the MVAM and the traditional constrained method, especially in the case of low SNR. Comparing the standard deviation and RMSE at each SNR scenario, we can tell that all these three methods give approximately unbiased estimates of the target direction. Additionally, the theoretical standard deviation calculated by (48) predicts the simulation results well. However, at low SNR, due to the disturbance of noise and a finite number of samples, the simulation results slightly deviate from the theoretical curve.

**Figure 4:**
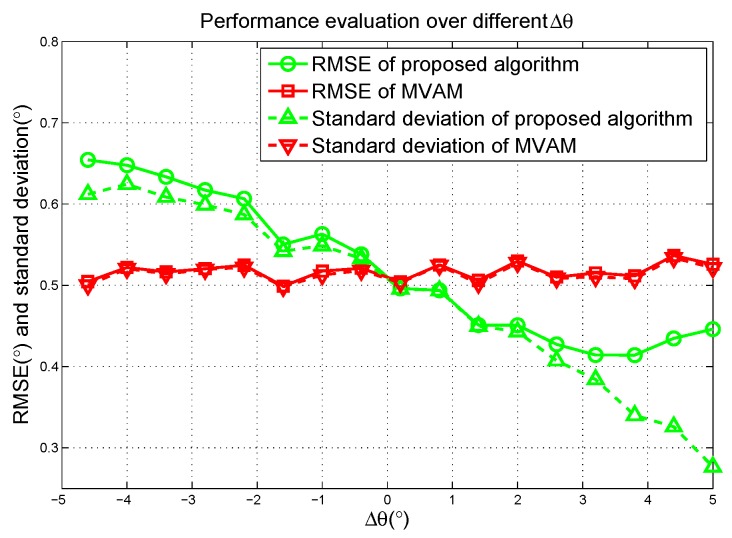
Root-Mean-Square Errors (RMSE) versus the deviation of the angle of target in scenario 2 of the linear array simulation.

The effect of choosing different Δθ is evaluated in Figure 4 where the MVAM is used as a comparison. SNR is set to be 10 dB. It can be seen that the RMSE of the proposed algorithm slowly decreases with Δθ growing until Δθ reaches half of the beamwidth and the RMSE near 0 is approximately equal to that of the MVAM. This result shows that by optimizing Δθ, the proposed algorithm can achieve better performance than the MVAM. It can also be observed that the standard deviation continues to decrease with Δθ growing beyond the half of beamwidth which is analyzed in section III. However, the RMSE deteriorates after that point which suggests that, in this case, the bias of the estimation cannot be ignored.

**Figure 5:**
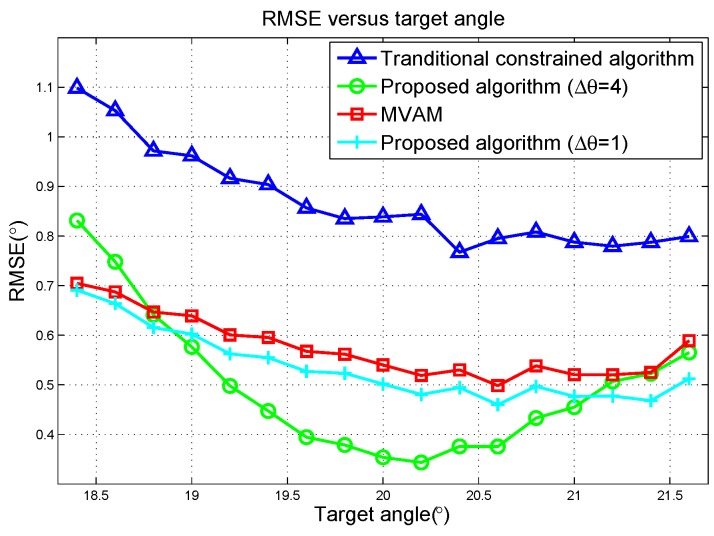
Antenna placement of the planar array.

Finally, the RMSE versus target angle is plotted in Figure 5. When the target is very close to the radar look direction, the proposed algorithm with Δθ=4 achieves much better performance than the MVAM and the traditional constrained method. However, its performance deteriorates fast with the target offset angle growing and would be worse than the MVAM when the offset angle continues growing. This deterioration is due to the bias increase of the estimation. As can be seen in Figure 5, when Δθ is adjusted to 1, the proposed algorithm has better performance than the MVAM at every offset angle of target. However, compared with the case where Δθ=4, its RMSE is worse at positions close to the radar look direction.

From the above simulation results, we can see that the proposed algorithm always outperforms the traditional constrained method and when Δθ goes to 0, it gives the same performance as the MVAM. With proper choice of Δθ, the proposed algorithm can outperform the MVAM.

### 5.2. Simulation in Planar Array

In this section, a simulation is conducted based on a 12×12 rectangular planar array with elements horizontally and vertically spaced by half of the wavelength. This whole array is divided into 16 3×3 sub-arrays all steered to look at the direction $\left(70^{\circ },90^{\circ }\right)$. For simplicity of comparison and discussion, no amplitude weighting is considered at the array elements. The target is located at $\left(69^{\circ },89^{\circ }\right)$ with power equal to the internal noise. Two noise jammers with power of 30 dB relative to the internal noise are incident from the direction $\left(77^{\circ },90^{\circ }\right)$ near the mainlobe and the direction $\left(84^{\circ },90^{\circ }\right)$ in the sidelobe region. The time series data is passed through a 100-point fast Fourier transform (FFT) filter before applying the adaptive weighting. The covariance matrix R is estimated from 100 snapshots.

**Figure 6:**
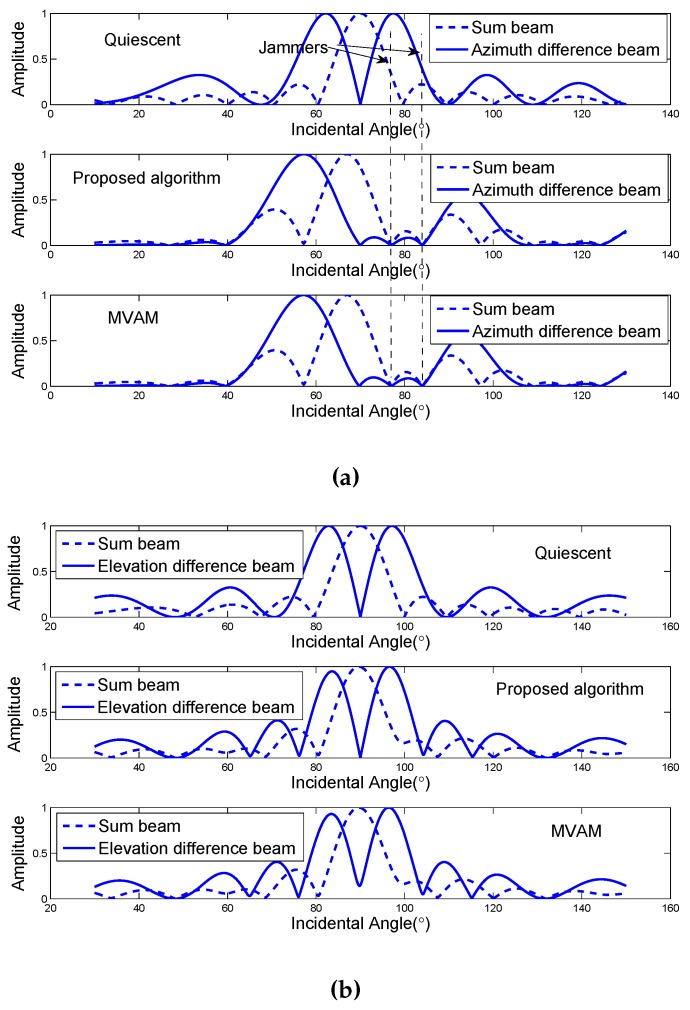
Azimuth and elevation beam pattern cuts. (**a**) Azimuth difference beam pattern; (**b**) Elevation difference beam pattern.

In the first scenario, instead of solving (35), we first evaluate two choices of Δθ: $\Delta \theta =3^{\circ }$ and $\Delta \theta =-3^{\circ }$, whose absolute value is almost half of the beamwidth. The other parameter Δφ is chosen to be 0.1. The azimuth and elevation difference beam pattern cuts for the case of $\Delta \theta =-3^{\circ }$ are plotted in Figure 6. It can be observed that nulls are placed at the directions of jammers for both the proposed algorithm and the MVAM and like the case of linear array, the MVAM tends to place a null at the radar look direction.

**Figure 7:**
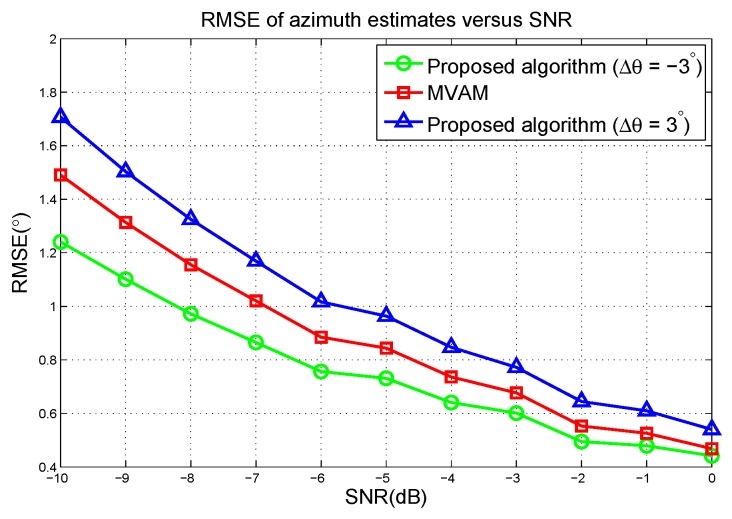
RMSE of azimuth estimates versus the SNR in the first scenario.

The RMSE of azimuth estimates versus the SNR are plotted in Figure 7. It can be seen that the proposed algorithm outperforms the MVAM with $\Delta \theta =-3^{\circ }$ but performs worse than the MVAM in the case of $\Delta \theta =3^{\circ }$. Therefore, to achieve the best performance, the parameters need to be optimized. Combining with the simulation results in the case of linear array, it can be observed that in all these scenarios, the proposed algorithm with optimized parameters outperforms the MVAM.

**Figure 8:**
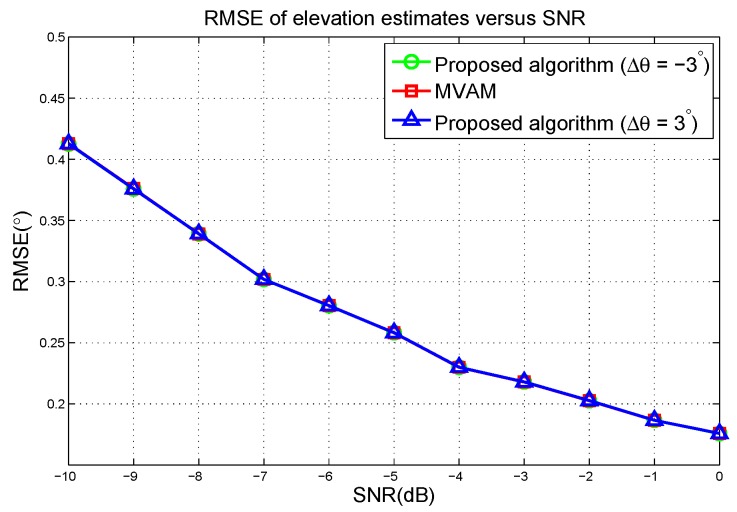
RMSE of elevation estimates versus the SNR in the first scenario.

The RMSE of elevation estimates versus the SNR are plotted in Figure 8. From Figure 8, we can see that the proposed algorithm and the MVAM have the same performance at each SNR. Therefore, we demonstrate that by choosing Δφ close to 0, the proposed algorithm gives the same performance as the MVAM.

**Figure 9:**
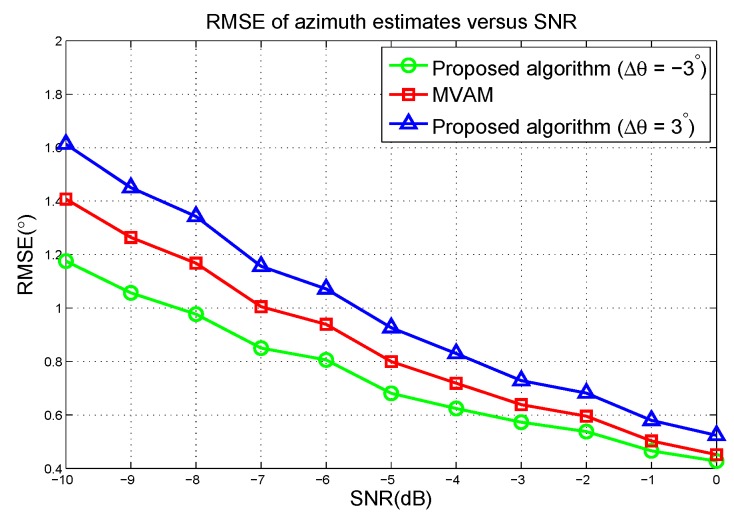
RMSE of azimuth estimates versus the SNR in the second scenario.

**Figure 10:**
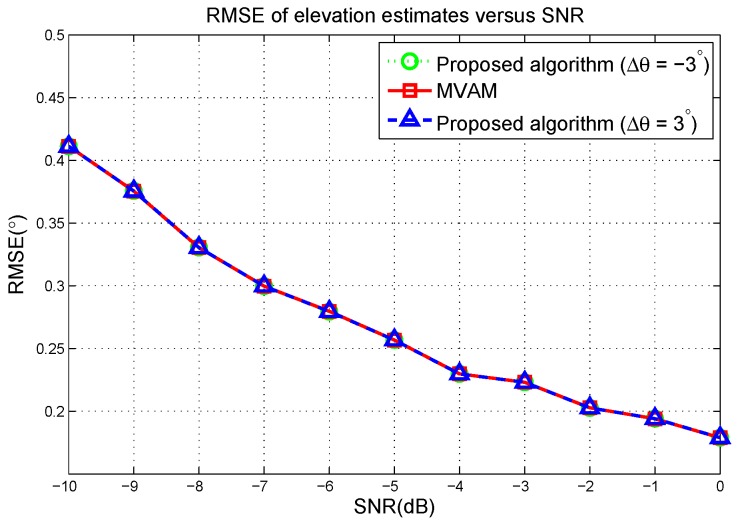
RMSE of elevation estimates versus the SNR in the second scenario.

In the second scenario, we evaluate the performance w.r.t. different target locations. Assume the target is incident from the direction $\left(71^{\circ },91^{\circ }\right)$ while the rest of the conditions remain the same as the first scenario. Again, two sets of parameters $\Delta \theta =3^{\circ },\Delta \varphi =0.1^{\circ }$ and $\Delta \theta =-3^{\circ },\Delta \varphi =0.1^{\circ }$ are evaluated. The RMSE of estimates versus the SNR are plotted in Figure 9 and Figure 10. It can be seen from Figure 9 that in the estimation of azimuth, the proposed algorithm with $\Delta \theta =-3^{\circ }$ still outperforms the MVAM. In Figure 10, it can be seen that the estimation of elevation is irrelevant with the choice of Δθ and with $\Delta \varphi =0.1^{\circ }$, the proposed algorithm is equivalent with the MVAM. More results with different target locations are shown in Table 1 and Table 2. In these cases, the SNR is fixed at −6 dB. From case 1 to 4, the target is incident from $\left(71^{\circ },89^{\circ }\right)$, $\left(69^{\circ },91^{\circ }\right)$, $\left(72^{\circ },92^{\circ }\right)$ and $\left(68^{\circ },88^{\circ }\right)$ respectively.

In the third scenario, we evaluate the performance of the proposed algorithm with different jammer locations. The target is located at $\left(69^{\circ },89^{\circ }\right)$ with the SNR being −5 dB. We consider three different cases. In the first case, assume two jammers are incident from $\left(77^{\circ },90^{\circ }\right)$ and $\left(70^{\circ },97^{\circ }\right)$ respectively. In the second case, they are $\left(63^{\circ },90^{\circ }\right)$, $\left(56^{\circ },90^{\circ }\right)$ and in the third case, $\left(63^{\circ },90^{\circ }\right)$, $\left(70^{\circ },83^{\circ }\right)$. The rest of the conditions are the same as scenario one. The RMSE of the proposed algorithm and the MVAM are shown in Table 3 and Table 4. For the proposed algorithm, parameters are determined by solving (35) with grid search within 3 dB beamwidth around the radar look direction. The weighting matrix K is chosen to be the unity matrix. Since the range of searching area is small, the computation cost is reasonable. For case 1, the optimum parameters are $\Delta \theta =-3^{\circ }$ and $\Delta \varphi =-3^{\circ }$. For case 2, the optimum parameters are $\Delta \theta =3^{\circ }$ and $\Delta \varphi =-3^{\circ }$ while for case 3, they are $\Delta \theta =3^{\circ }$ and $\Delta \varphi =3^{\circ }$. It can be seen that the proposed algorithm outperforms the MVAM in all three cases. However, the performance of the proposed algorithm depends on the locations of jammers. In the case where both jammers have the same elevation angle (case 2), the advantage of the proposed algorithm is more obvious than the other cases.

## 6. Conclusions

In this paper, a novel constrained monopulse algorithm has been proposed for phased array radar. In the real battlefield, airborne target signals are usually masked by strong noise jammers which are close to the target in space. By using novel adaptive difference beam weights, the difference beam adaptively provides suppression against unwanted signals and maintains the beam shape around the look direction. Therefore, the monopulse ratio formed by the proposed algorithm is able to maintain the linearity around the look direction and minimize the effect from noise jamming. The theoretical mean and variance of the proposed monopulse estimator is derived for performance evaluation. From the theoretical analysis and mathematical simulation, it can be seen that the estimation of the target’s direction is unbiased and accurate. In comparison with the previous methods, it is found that the MVAM is equivalent with a special case of the proposed algorithm where the constrained point is chosen to be close to the look direction. By optimizing the constrained point, the proposed algorithm can outperform the existing adaptive monopulse techniques. The optimized constrained point is derived in the linear array case. In the planar array case, the optimization requires recursive iteration, thus is not suggested for real-time application. However, in such case, the proposed algorithm can still achieve the same performance as the MVAM by making the constrained points close to the look direction. Mathematical simulation results in scenarios where severe jamming sources occur near the mainlobe, demonstrate that the proposed monopulse technique can accurately acquire target direction and outperform the previous adaptive monopulse techniques. In addition, the computational complexity of the proposed algorithm is found to be $O\left(l^{2}\right)$ which is about the same as the traditional adaptive monopulse techniques and usually affordable in modern phased array radar. All in all, the proposed algorithm proves to be effective in the real scenarios where strong jammers occur near the target.

## Figures and Tables

**Table 1:** RMSE of azimuth estimates versus target locations.

Method	Case 1	Case 2	Case 3	Case 4
Proposed algorithm	$0.79^{\circ }$	$0.60^{\circ }$	$1.10^{\circ }$	$0.64^{\circ }$
MVAM	$0.93^{\circ }$	$0.72^{\circ }$	$1.20^{\circ }$	$0.79^{\circ }$

**Table 2:** RMSE of elevation estimates versus target locations.

Method	Case 1	Case 2	Case 3	Case 4
Proposed algorithm	$0.27^{\circ }$	$0.22^{\circ }$	$0.72^{\circ }$	$0.37^{\circ }$
MVAM	$0.27^{\circ }$	$0.22^{\circ }$	$0.72^{\circ }$	$0.37^{\circ }$

**Table 3:** RMSE of azimuth estimates versus jammer locations.

Method	Case 1	Case 2	Case 3
Proposed algorithm	$0.33^{\circ }$	$0.81^{\circ }$	$0.42^{\circ }$
MVAM	$0.33^{\circ }$	$0.96^{\circ }$	$0.47^{\circ }$

**Table 4:** RMSE of elevation estimates versus jammer locations.

Method	Case 1	Case 2	Case 3
Proposed algorithm	$0.32^{\circ }$	$0.21^{\circ }$	$0.37^{\circ }$
MVAM	$0.33^{\circ }$	$0.28^{\circ }$	$0.40^{\circ }$
